# Can small drugs predict the intrinsic aqueous solubility of ‘beyond Rule of 5’ big drugs?

**DOI:** 10.5599/admet.794

**Published:** 2020-04-25

**Authors:** Alex Avdeef, Manfred Kansy

**Affiliations:** 1 in-ADME Research, 1732 First Avenue #102, New York, NY 10128 USA; 2 79111 Freiburg im Breisgau, Germany

**Keywords:** aqueous intrinsic solubility, Rule of 5 (Ro5), beyond Ro5 (bRo5), General Solubility Equation (GSE), Abraham Solvation Equation (ABSOLV), Partial Least Squares (PLS), Random Forest regression (RFR), intramolecular hydrogen bonding (IMHB), Solubility Enhancement–Big Molecules (*SEBM*)

## Abstract

The aim of the study was to explore to what extent small molecules (mostly from the Rule of 5 chemical space) can be used to predict the intrinsic aqueous solubility, S_0_, of big molecules from beyond the Rule of 5 (bRo5) space. It was demonstrated that the General Solubility Equation (GSE) and the Abraham Solvation Equation (ABSOLV) underpredict solubility in systematic but slightly ways. The Random Forest regression (RFR) method predicts solubility more accurately, albeit in the manner of a ‘black box.’ It was discovered that the GSE improves considerably in the case of big molecules when the coefficient of the log P term (octanol-water partition coefficient) in the equation is set to -0.4 instead of the traditional -1 value. The traditional GSE underpredicts solubility for molecules with experimental S_0_ < 50 μM. In contrast, the ABSOLV equation (trained with small molecules) underpredicts the solubility of big molecules in all cases tested. It was found that the errors in the ABSOLV-predicted solubilities of big molecules correlate linearly with the number of rotatable bonds, which suggests that flexibility may be an important factor in differentiating solubility of small from big molecules. Notably, most of the 31 big molecules considered have negative enthalpy of solution: these big molecules become less soluble with increasing temperature, which is compatible with ‘molecular chameleon’ behavior associated with intramolecular hydrogen bonding. The X-ray structures of many of these molecules reveal void spaces in their crystal lattices large enough to accommodate many water molecules when such solids are in contact with aqueous media. The water sorbed into crystals suspended in aqueous solution may enhance solubility by way of intra-lattice solute-water interactions involving the numerous H-bond acceptors in the big molecules studied. A ‘Solubility Enhancement–Big Molecules’ index was defined, which embodies many of the above findings.

## Introduction

The aqueous solubility of compounds is an important physical property to assess in pharmaceutical research and development [1-4]. Solubility of potentially promising compounds not yet synthesized may be estimated computationally. Many methods for predicting solubility have been described [5-7], based on linear quantitative structure-property relationship (QSPR) approaches [8-16]. More recent methods have evolved using machine learning statistical methods [17-20]. The molecular descriptors needed for these predictions are most often calculated from two-dimensional (2D) structures.

In the early 1990s, attrition due to poor oral bioavailability and pharmacokinetics (PK) was responsible for nearly 40% of compounds being rejected in clinical studies [21]. Lipinski’s Rule of 5 (Ro5) emerged as part of the effort to address critical issues underlying the high attrition [1]. The Ro5 guidelines suggest that compounds are more likely to be orally bioavailable if three or more of these rules are adhered to: molecular weight, *M*_W_ ≤ 500 Da, calculated octanol-water partition coefficient, *clogP* ≤ 5, number of H-bond donors, *NHD* ≤ 5, and number of H-bond acceptors, *NHA* ≤ 10. High-throughput screening strategies of physicochemical properties of research compounds led to improvements. By the new millennium, attrition due to PK was reduced to below 10% [22].

However, many recently approved drugs are larger, more lipophilic, and possess more H-bond acceptors, compared to drugs in the Ro5 chemical space [22]. Many of the newly-approved therapeutics are used in immunosuppression, treatment of infectious/viral diseases, and in oncology. Since 2014, an increasing number of ‘beyond the Rule of 5’ (bRo5) commentaries have stressed that the strict adherence to the Ro5 may result in lost opportunities [21-30]. Cell-permeable and orally-bioavailable drugs can be discovered far into bRo5 chemical space. Some of these drugs are derived from natural products, which appear to be better suited for the newer targets which possess large and flat binding sites. Nevertheless, concerns have been raised over the expected higher pharmacokinetic risks from bRo5 compounds: low solubility, poor cell permeability, increased cellular efflux, and extensive metabolism. Medicinal chemists have applied tactics to lessen some of the risks: (a) reducing or shielding polarity by N-methylation, or by bulky side chains, (b) selecting compounds with flexible rings structures allowing for conformational lability, and (c) selecting compounds which can reversibly form multiple *intra*molecular H-bonds (IMHB) to shield polar groups during passage across lipoidal cell barriers, in the manner of ‘molecular chameleons’ [26-31].

Although most of the bRo5 commentaries have emphasized permeability, absorption, and potency topics, Bergström *et al*. [25] focused on solubility aspects and the promising computational biopharmaceutical modeling strategies to help identify ‘formulate-ability’ during lead optimization and early development stages of bRo5 compounds. Caron *et al.* [29] considered case studies of kinetic solubility (measured in pH 7.4 phosphate buffer containing 1-5% DMSO) of bRo5 molecules, in terms of the tendency to form IMHBs and their effect on solubility.

In our preceding study [20], three methods of solubility prediction were compared: (a) Yalkowsky’s General Solubility Equation (GSE) [8], (b) Abraham Solvation Equation (ABSOLV) [11], and (c) Random Forest regression (RFR) [19] statistical machine learning. The linear ABSOLV and the RFR multiple decision-tree methods were trained with molecules in the *Wiki-pS_0_* database. Thirty of the most important descriptors identified in the RFR analysis [20] were subjected to Principle Components Analysis (PCA). The scores plot had the appearance of a ‘comet’ – with a dense symmetrical core of Ro5 compounds about the origin of the first two principle components and a long sparsely-populated tail of big molecules queuing far into the *lower-right* quadrant. The molecules in the tail have high H-bond acceptor strength (*NHA*), topological polar surface area (*TPSA*), fraction of sp^3^ carbons (FractionCSP3), and possess *M*_W_ > 800 Da – many of the recognized hallmarks of bRo5 chemical space.

The aim of the present study was to explore to what extent small molecules (mostly Ro5) can be used to predict the intrinsic aqueous equilibrium solubility of big molecules (all bRo5 drugs), i.e., can the ‘head predict the tail’?

## Methods

### Computational models

Three computational approaches described below span from the theoretically sound and easy-to-use GSE, the sound and flexible ABSOLV, and the more accurate (but somewhat of a ‘black box’) RFR.

#### General Solubility Equation (GSE)

Expanding on the work of Irmann [32] and Hansch *et al.* [33], Yalkowsky and coworkers in 1980 developed and thereafter popularized the General Solubility Equation (GSE), to enable the prediction of the solubility of organic molecules in water [8, 9, 34-38]. Just two variables, melting point (*mp* in °C) and octanol-water partition coefficient, log *P*, are used in the equation to predict solubility (in log molar units):


(1)

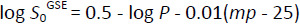



Below, the derivation of Eq. (1) is briefly reviewed in terms of its underpinning assumptions to determine if any are incompatible with bRo5 molecules. Also, the thermodynamics of solubility are well cast by Eq. (1), which can apply to all models tested here. It is useful to start by dissecting aqueous solubility in terms of the Gibbs free energy based on the thermodynamic fusion cycle. The dissolution of a non-ionized crystalline substance suspended in water can be viewed in terms of two major contributions: (a) *crystal lattice* – energy has to be provided to break down the lattice to form a hypothetical ‘supercooled’ liquid (sliq), and (b) *solvation* – energy is released when the liquefied solute dissolves in water. The total solubility of the solute in water is the product of the above two contributions (lattice and solvation), which in logarithmic terms can be stated as the sum [36, 37]:


(2)

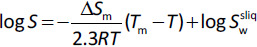



### Crystal lattice effect

The lattice contribution, first term on the right side of Eq. (2), arises from the application of the van’t Hoff equation, where ∆*S*_m_ (kJ/mol·K) is the entropy of melting (fusion) and *T*_m_ is the melting point (in K units). By the ‘Walden’s rule’ approximation [36, 37], ∆*S*_m_ = 0.0565 kJ/mol∙K for many organic compounds (*particularly for rigid planar molecules, but less so for spherical molecules*). At 25 °C, 2.3 *RT* = 5.706 kJ/mol∙K. On substituting these constants, Eq. (2) at 25 °C reduces to:


(3)

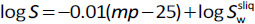



### Solvation effect

The solvation contribution, right-most term in Eq. (3), was investigated by Hansch and coworkers [33]. For 156 simple organic liquid solutes, they demonstrated that solubility correlated with octanol-water partition data as described by a Collander-like linear equation: log *S* = c_0_ + c_1_log *P*. Octanol, possessing nearly identical calculated H-bond donor and acceptor strength, was selected as a model organic solvent. For a series of aromatics, alkyl halides, and alkanes liquid solutes, c_0_ intercepts were calculated to be +0.34, +0.83, and -0.25, respectively. The derived c_1_ slope factors were -1.0 for aromatics, -1.22 for alkyl halides, and -1.24 for alkanes.

For a liquid solute, log *P* relates to the Gibbs free energy for the sum of solute-solute and water-water cohesive interactions minus twice the solute-water adhesive interactions [8]. For a liquid solute with the aqueous solubility of *S*_w_^liq^ and the solubility in octanol as *S*_oct_^liq^, it can be approximated that *P* = *S*_oct_^liq^ /*S*_w_^liq^ (*assuming activity equals concentration and that solute aggregates/micelles don’t form* [39]). It follows that if the slope c_1_ = -1, then the intercept c_0_ = log *S*_oct_^liq^.

Yalkowsky and coworkers surmised that c_0_ = 0.5, by the following reasoning. Entropy of mixing favors complete miscibility of the two liquids; *i.e*., the mole fraction = 0.5 (*8*). (*This is likely to be valid for apolar* [37], *but may not be accurate for large polar molecules like those found in the bRo5 chemical space.*) Since the molar concentration of pure octanol is 6.32 M, the log *S*_oct_^sliq^ = log (6.32x0.5) = 0.5 (*assuming the solute liquid density is near that of octanol*). On rearranging the log form of *P* defined as the solubility ratio, log *S*_w_^sliq^ = 0.50 – log *P*. On substitution of the latter term into Eq. (3), the GSE, Eq. (1), is so derived.

The above considerations suggest that Eq. (1) may have possible limitations in bRo5 chemical space: (i) Lattice energy of rigid-planar molecules may be different from those of spherical molecules; (ii) Octanol as the model for the supercooled liquid solute may not be accurate for large polar or conformation-flexible molecules; (iii) Non-ideal activity may arise due to solute self-aggregation (e.g., dimer formation of vancomycin), possible micellization (e.g., ubiquinone, iodoxamic acid), and ‘molecular chameleon’ IMHB effects [29, 31].

### Using Calculated log P (clogP)

The original two variables (*mp*, log *P*) were taken to be experimental values. In a pharmaceutical research setting, such experimental values may not be available in early studies. It has become a common practice to use calculated values, *clogP*, in place of measured log *P* in Eq. (1). Apparently, the accuracy of GSE lessens, but only slightly. The use of calculated *mp* is less frequent, since the accuracy of such predicted values is thought to be relatively low. In the present investigation, experimental values were applied when available, and were calculated in a small number of instances [40].

#### Abraham Solvation Equation (ABSOLV)

Abraham and Le [11] amended the Abraham Solvation Equation [41] to predict intrinsic solubility (log molar):


(4)

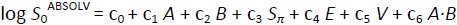



The independent variables are the five solute descriptors accounting for the transfer of solute from one phase to another: *A* is the sum of H-bond acidity (similar to *NHD*), *B* is the sum of H-bond basicity (similar to *NHA*), *S*_π_ is the dipolarity/polarizability (subscripted here, so as not to be confused with solubility), *E* is an excess molar refraction in units of (cm^3^∙mol^-1^)/10, and *V* is the McGowan characteristic volume in units of (cm^3^∙mol^-1^)/100.

In principle, the five solute variables could account for any shortcomings of just using *clogP*, as in Eq. (1). The *A∙B* cross-term in Eq. (4) was intended to address *inter*molecular H-bond interactions between acid and base functional groups in the solid or liquid environment. Its inclusion, as an alternative to using the *mp* term in Eq. (1), was intended to improve the prediction accuracy of Eq. (4).

The c_0_-c_6_ coefficients in Eq. (4) are usually determined by multiple linear regression (MLR), trained on a set of intrinsic solubility values of a diverse collection of molecules. The five Abraham solvation descriptors may be calculated from 2D structure (introduced as a SMILES text or as coordinates in a ‘mol’ format) using the program ABSOLV [42] (*cf*., www.acdlabs.com). In the present study, the seven MLR coefficients were re-determined using our own training data (*Wiki-pS_0_* database), with log *S_0_* data weighted in the regression analysis according to estimated measurement errors [20].

Furthermore, we attempted to improve the accuracy of Eq. (4) when applied to big compounds, by introducing a nonlinear term,


(5)

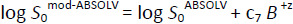



Due to potentially high linear correlations among the descriptors, partial least squares (PLS) regression (open source package in R: https://cran.r-project.org/web/packages/pls/) was used (instead of MLR) to determine c_0_-c_7_ for given values of z. Several z values in the range 0.9 to 2.0 were tested; the best-fit exponent was selected as the minimum point in the fit of PLS root-mean-square error (RMSE) vs. z.

#### Random Forest regression

Of the new machine-learning statistical approaches, the Random Forest regression (RFR) method is thought to be one of the most accurate in predicting solubility [17-20]. RFR can be employed ‘off the shelf,’ requiring only minimal learning [19]. The provided default ‘tuning’ parameters are nearly optimal. However, its ‘black box’ nature makes the outcome of the analysis challenging to interpret in terms of the descriptors used, even when the most important descriptors are quantitatively ranked in RFR.

The method was introduced in 2001 by Brieman [43], and is implemented in the open-source ‘randomForest’ library for the R statistical software [44-46]. RFR works by constructing an ensemble of hundreds of decision trees [47]. The tutorial chapter by Walters [19] is highly recommended as a means to get started with RFR.

The first applications of RFR to predict solubility appeared in 2007 [17, 48]. Schroeter *et al.* [48] used *S*_w_ and *S*_pH_ data to train a RFR method, using ~4000 measurements mostly taken from secondary sources [12, 49, 50] and some from in-house (Bayer Schering Pharma) sources. For the Huuskonen data [12] as test set, RMSE = 0.66 log unit (n=1290) was reported. For the solubility data in the domain of applicability (DOA) matching that of research compounds (10^-3^ to 10^-7^ M solubility), the RFR method indicated RMSE ~ 0.85 log.

In the Palmer *et al.* [17] RFR study, aqueous solubility values of 998 structurally diverse druglike solid organic compounds were gathered from similar secondary sources [12, 51, 52]. The authors used the Molecular Operating Environment (MOE) [53] to generate 126 2D (*clogP*, *MR*, charged-surface properties, atom, group, and H-bond counts, connectivity and topological indices) and 36 3D (total potential energy, electrostatic contributions, molecular shape, and solvent-accessible surface area) descriptors. Randomly splitting the entire data into a training set (70%) and an internal validation set (30%) produces a good measure of the model predictivity of compounds not included in the training set: r^2^ = 0.89, RMSE = 0.69 log, n = 330.

More recently, Walters [19] critically compared the Huuskonen thermodynamic *S*_w_ values (n = 1274) [12], the Llinas *et al.* thermodynamic *S*_0_ values (n = 94) [54] and PubChem (n=1000) kinetic high-throughput solubility [55] databases using the RFR framework. Avdeef [20] applied RFR, trained with 6355 log *S_0_* values, to predict the solubility of four well-publicized small external test sets, occasioning in RMSE from 0.66 to 1.05 log.

## Data

### Wiki-pS_0_ database

The intrinsic aqueous solubility database *Wiki-pS_0_* (*in-ADME* Research) [20, 56] was used. It now contains 6473 log *S*_0_ (log molar) entries, based on measured aqueous solubility values of 3065 different compounds (excluding agrochemicals) collected from 1415 cited references. The most reliable published data had been determined by the saturation shake-flask (SSF) method, particularly when measured *as a function of pH*. In the majority of the cases, the literature data were further processed, using *p*DISOL-X (*in-ADME* Research) [56-61], to extract intrinsic solubility (*S*_0_) values from reported aqueous free-acid/base or salt solubilities (*S*_w_), or solubilities at specified pH (*S*_pH_), or log *S*-pH profiles. All of the molecules are solids at room temperature (except propofol). There are 1127 log *S*_0_ entries derived from 10167 individual-pH log *S* measurements. About half of the data sources originate from secondary listings and the rest are from primary sources. In the case of secondary sources, the citations to the original work were generally available, and in many cases were consulted for clarifications. Melting points are included in the database. When measured *mp* were not found (19% of entries), *mp* were calculated by the Lang and Bradley method [40] in the QsarDB open repository of data and prediction programs (http://qsardb.org/repository/predictor/10967/104?model=rf).

### Physicochemical properties of the big molecules

**Figure 1.**
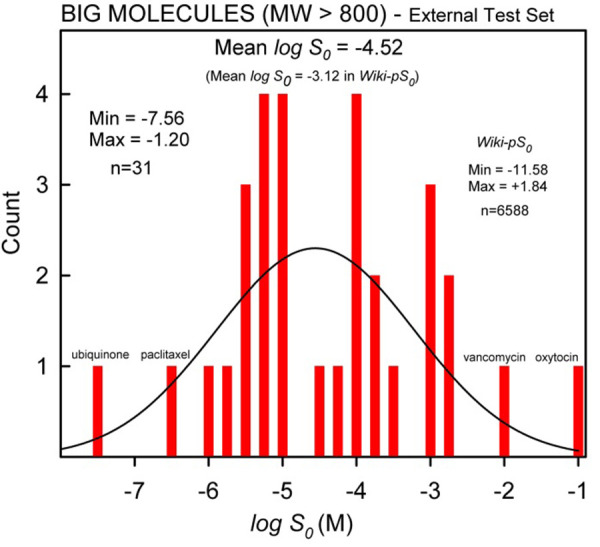
Distribution of the big-molecule intrinsic aqueous solubility values in Wiki-pS0

In this study, the compounds in *Wiki-pS_0_* were divided into two groups: ‘big’ (*M*_W_ ≥ 800 Da, structures in **Appendix**) and ‘small’ molecules. The demarcation was motivated by the shape of the principle components scores plot (Fig. 10 in [20]). There are 31 molecules (58 log *S*_0_ entries) in the ‘big’ set. Table 1 lists their characteristic properties. Figure 1 shows the distribution of big-molecule log *S*_0_ values. On the average, the big molecules are less soluble (-4.52) than the small molecules (-3.12).

**Figure 2.**
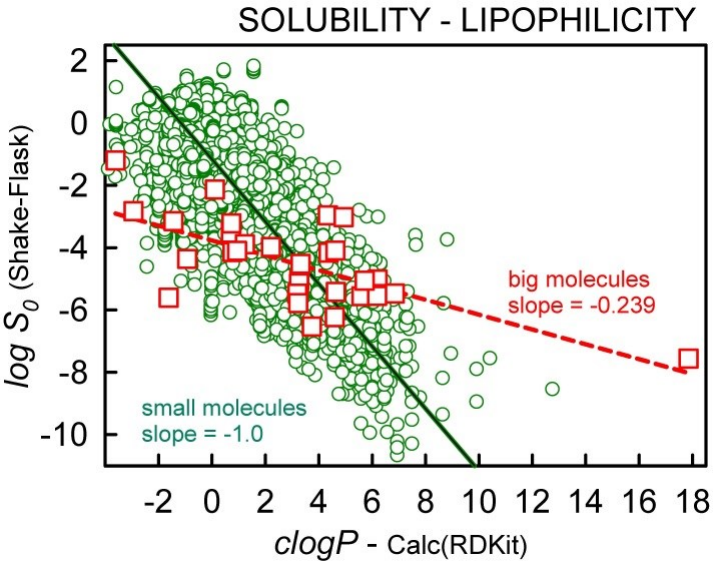
Plot of log S0 versus octanol-water partition coefficient, clogP, calculated using the RDKit software [62]. Squares refer to big molecules; circles refer to small molecules

Figure 2 shows the trend between measured log *S_0_* and *clogP* (calculated in RDKit [62]: Wildman-Crippen sum of atomic contributions – cf., *Abbreviations and definitions*) for the two groups of molecules. The scatter is substantial. Nevertheless, the small molecules (green circles) show the expected -1 slope, whereas the big molecules (red squares) show an apparent slope of -0.239. This is an important characteristic differentiating the two groups.

### Characteristics of the big molecules

**Figure 3.**
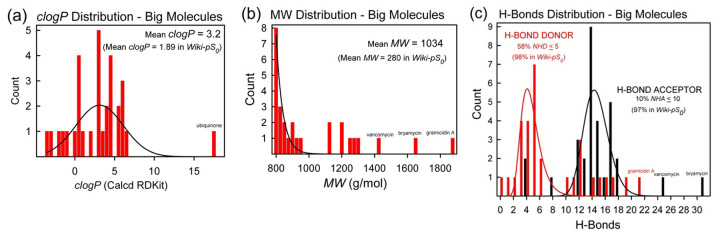
Big-molecule property distributions: **(a)** *clogP*, **(b)** molecular weight (*M*_W_), and **(c)** number of H-bond donors (*NHD*) and acceptors (*NHA*). The separation between the groups is greater than that found in small molecules [20].

Figure 3 shows property distributions as possible indicators of bRo5 ‘big-drug-likeness.’ Frame (a) shows the *clogP* distribution: on the average, *clogP* of the ‘big’ set (3.17) is greater than that of the ‘small’ set (1.89) [20]. Frame (b) shows the distribution of molecular weights about the mean value 1034 Da (compared to 280 Da in the entire set [20]). Frame (c) considers H-bonding characteristics. The red bars (tallest near 5) refer to H-bond donor counts (*NHD*). The black bars (tallest near 15) refer to H-bond acceptors (*NHA*). In the small-molecule set, the *NHA* and *NHD* groups overlap considerably, as illustrated elsewhere [20]. But, in the big-molecule set (Fig. 3c, Table 1) the *NHA* and *NHD* distributions are wider apart: the acceptor count increases, but not so much the donor count. This is an important characteristic differentiating the big-small molecule groups.

## Results and discussion

### GSE applied to big compounds

#### Hydrophilicity (*clogP*) effect in big lipophilic molecules

The linear dependence of log *S*_0_ on *clogP* (Fig. 2) was further analyzed in the context of Eq. (1). MLR of the SD-weighted log *S*_0_ data confirmed the large difference in *clogP* contributions in the two groups of molecules:


(6a)

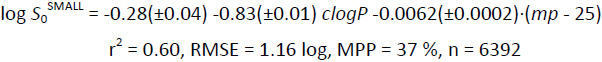




(6b)

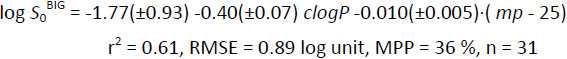



The extent of ‘correct’ predictions is defined here by MPP (measure of prediction performance: percentage of the absolute residuals ≤ 0.5 log unit).

Apparently, *crystal lattice* contributions are not appreciably different in the two groups of molecules; the refined temperature coefficients in the two sets are close to the GSE value (-0.01) in Eq. (1). Hence, solution-phase interactions appear to dominate solubility [98].

The intercept constants suggest that big-molecule ‘supercooled’ liquid solutes are less miscible in octanol by 1-2 orders of magnitude than suggested by the original Yalkowsky analysis [8, 37]. The intercepts in Eqs. (6a) and (6b) are nearer to those of alkane solutes found in the Hansch *et al.* [33] study, compared to the constant in Eq. (1). For the big molecules, the highly negative intercept (*i.e*., *decreased* solubility of the supercooled liquid in the octanol phase) depresses the solute water solubility by a constant amount.

Countering that, the -0.4 slope factor lessens the contribution of lipophilicity to the calculated solubility of big molecules. The net result is that the traditional GSE overpredicts *S*_0_ for big molecules with experimental solubility above ~50 μM *(e.g., oxytocin, nafarelin)*, and underpredicts *S*_0_ below the crossover point *(e.g., everolimus, telithromycin).*

#### General Solubility Equation (GSE)

**Figure 4.**
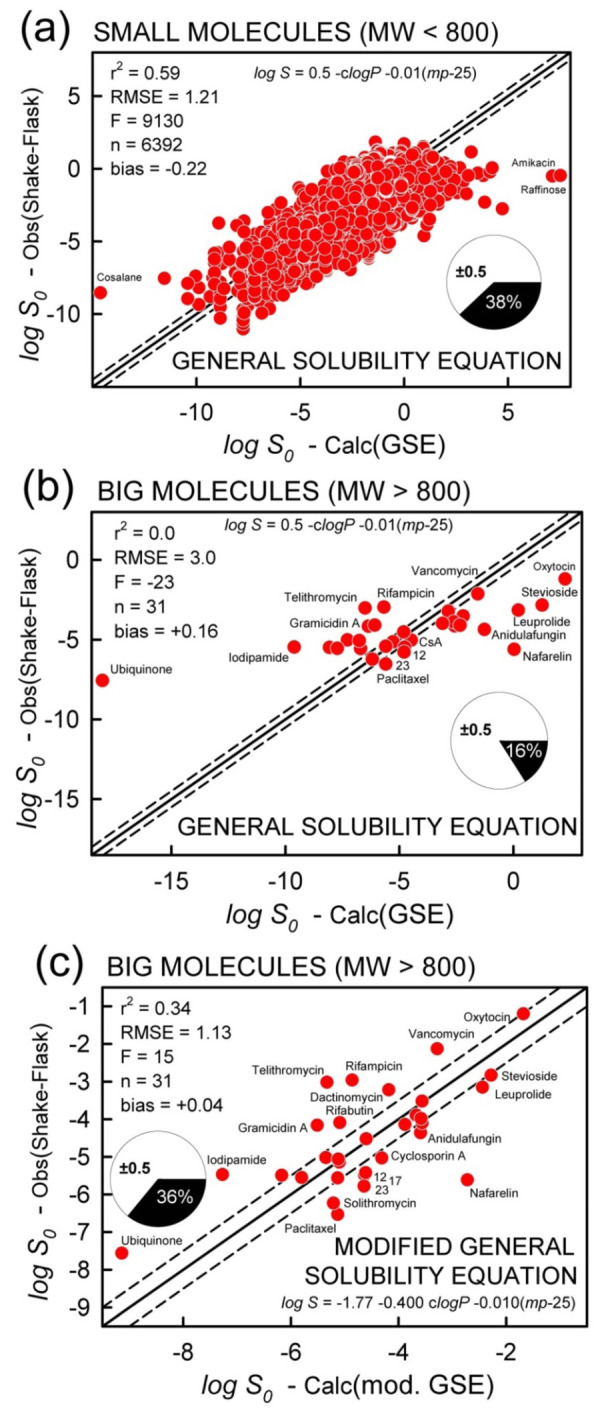
The log P in the figure refers to calculated octanol-water partition coefficients, clogP. The solid diagonals are the identity lines, and the dashed lines refer to ±0.5 log deviations. The MPP pie charts refer to percentage of ‘correct’ prediction, based on absolute residuals being ≤ 0.5 log. The prediction of log S0 values of (a) small molecules and (b) big molecules using the classical General Solubility Equation (numeric compound labels are of paclitaxel analogs). (c) When using just the big-molecule data, the three constants in the GSE (Eq. 1) subjected to MLR analysis (cf., Eq. 6b) produce the modified GSE, which is valid only for molecules with MW > 800 Da. There are not enough big molecules in the Wiki pS0 database to test the predictiveness of Eq. (6b).

Figure 4a shows the relationship between the measured solubility of small molecules and that calculated by the classic (‘untrained’) GSE. (Permanently-charged quaternary amines and big molecules are excluded in the training.) The r^2^, RMSE, MPP statistics are nearly identical to those associated with Eq. (6a), suggesting that ‘training’ does not improve the GSE predictivity for small molecules.

However, the performance of the ‘untrained’ GSE degrades when the equation is applied to the big molecules, as shown in Figure 4b, with r^2^ = 0.0, RMSE = 3.0 log (2.3 without ubiquinone), and MPP = 16%.

Figure 4c plots the big-molecule ‘trained’ GSE result (*cf*., Eq. 6b). *Note that this is not the equivalent of Ro5 molecules predicting the solubility of bRo5 molecules.* Rather, it highlights the hydrophilicity solvation effect of big molecules discussed in the preceding section. The traditional GSE requires adjustments when it comes to predicting the solubility of big molecules (*cf*., Fig. 4b).

### Abraham solvation model (ABSOLV) – weighted MLR to predict solubility of big compounds

#### Abraham linear equation for solubility prediction

The ABSOLV MLR analysis of the small-molecule data, weighted according to the estimated errors in the measured log *S_0_* values produced the equation


(7)

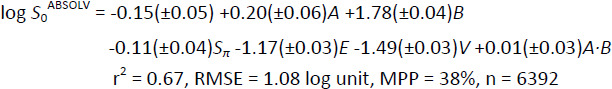



**Figure 5.**
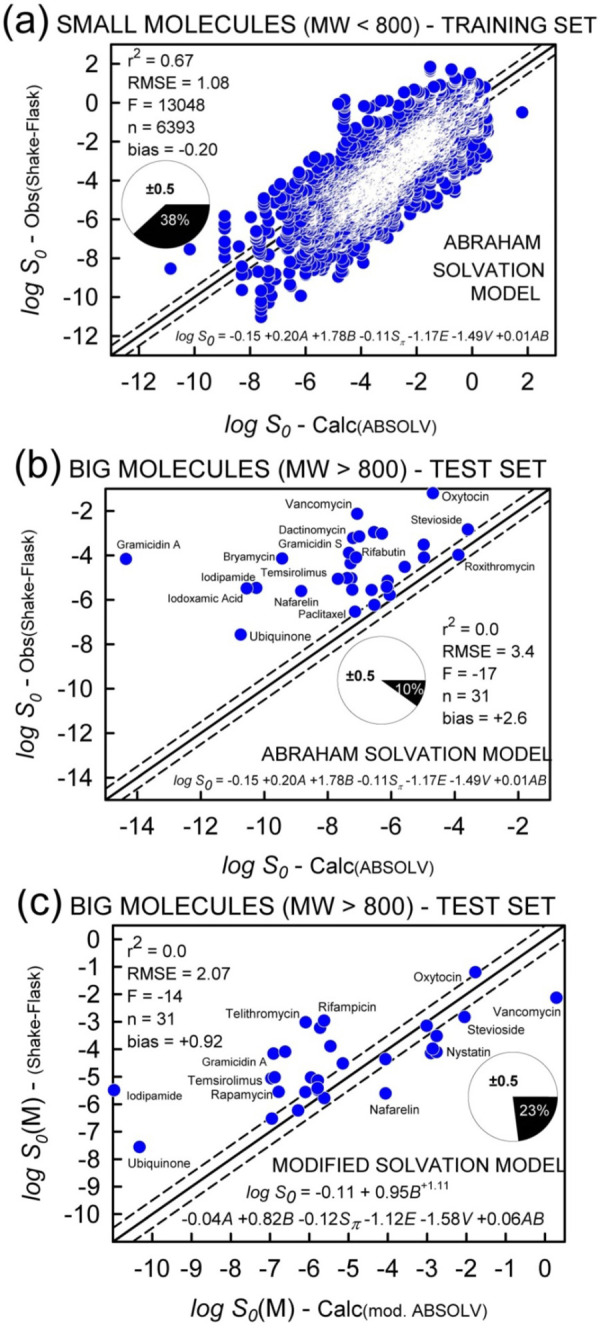
The prediction of log S0 values of (a) small molecules and (b) big molecules using the Abraham Solvation Equation (ABSOLV). (c) An additional nonlinear descriptor was added to the ABSOLV equation (cf., Eq. 5), which was then trained with the small-molecule set. This improved the prediction accuracy of the modified ABSOLV equation. The pie chart denotes MPP, the fraction of ‘correctly’ predicted molecules (absolute residuals ≤ 0.5 log unit).

The plot of measured log *S*_0_ as a function of the calculated values according to Eq. (7) is shown in Figure 5a. This trained ABSOLV model only slightly outperforms the small-molecule untrained/trained GSE model (Fig. 5a/Eq. 7 compared to Fig. 4a/Eq. 6a). The *A·B* cross term contribution appears to be negligible.

The application of Eq. (7) to the big-molecule set produced unidirectionally-skewed plot, as shown in Figure 5b. According to the ABSOLV model trained on small molecules, *the solubility of all big molecules is underpredicted.* For example, the gramicidin A measured log *S*_0_ = -4.16 ±0.41 is underestimated by 10 orders of magnitude. Vancomycin is underestimated by nearly 5 orders of magnitude.

An effort was made to improve the fit. A distinguishing characteristic of big compounds is that they contain a high level of H-bond *basicity* (*B*) character (Table 1). We tested several nonlinear contributions of the *B* descriptor, with the aim of amplifying its uniquely high positive impact on solubility (Eq. 7). In order to avoid difficulties due to descriptor correlations, PLS regression was used in place of MLR. The modified model, depicted in Figure 5c, is the best improvement that was found. The modified solvation model consisted of an additional nonlinear term, *B*^+z^, with z > 1. The best-fit value of z was determined to be 1.11. This new descriptor was expected to amplify the positive H-bond acceptor contribution in Eq. (7) in the case of big molecules. Other modifications were explored, but only the latter descriptor appeared to improve ABSOLV to a level slightly better than that of the classic GSE (Fig. 4b).

On inspection, the systematic errors in Figure 5b were found to correlate with the number of rotatable bonds (*nROT*): log *S*_0_^Obs^ – log *S*_0_^ABSOLV^ = 0.75 + 0.13 *nROT*, with r^2^ = 0.44 and RMSE = 1.62. Adding 0.75 + 0.13 *nROT* to Eq. (7) reduced the RMSE from 3.4 to 1.6 and the bias from 2.6 to 0.13 (r^2^ remained unchanged). However, this did not result in a significantly improved training-set model when *nROT* was added to the list of ABSOLV descriptors in a repeated PLS analysis. Flexibility appears to be important, but *nROT* is not significantly predictive in the *training* process. Caron *et al.* [30] demonstrated that *nROT* may have limitations because it neglects the contribution to flexibility from cyclic fragments in big molecules.

### Random forest regression using RDKit combined with Abraham descriptors and melting points

#### Descriptors

For the RFR model building, the 190 RDKit [62] descriptors (excluding those which were zero for all compounds) were combined with the *mp* and the ABSOLV descriptors. The *Abbreviations and definitions* section below identifies and defines the most important descriptors used in the RFR algorithm.

#### Training set and internal validation

**Figure 6.**
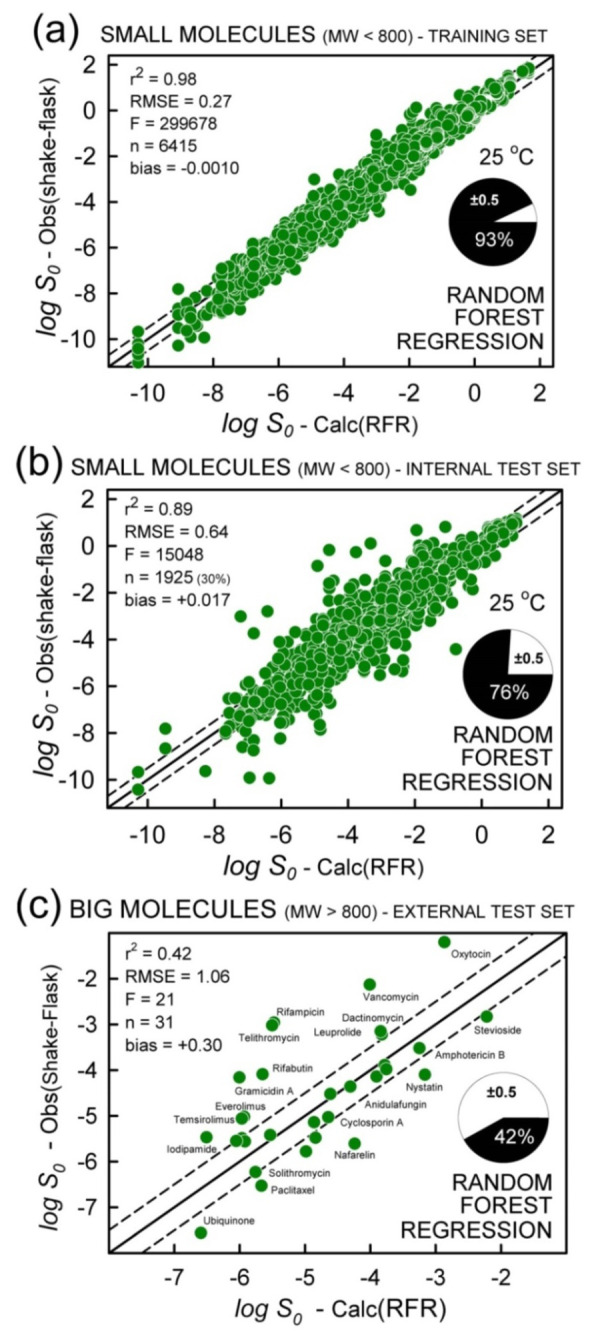
Random Forest regression analysis. (a) Training set using the small molecules. (b) Internal validation test set, based on 30% of the small molecules randomly selected. (c) External test set prediction of big molecules, not used in the method training.

Figure 6a shows the small-molecule training-set RFR analysis, resulting in the metrics: r^2^ = 0.98, RMSE = 0.27 log, bias = -0.001. This quality of fit indicates how well the model can incorporate the information presented by the descriptors and relate it to solubility in the training set [18]. The internal validation set of 1925 small-molecule solubility values (30%), randomly selected by the method, better indicates the ability of RFR to predict external test compounds which are unknown to the training process. Figure 6b shows the *internal* validation test set prediction results: r^2^ = 0.89, RMSE = 0.64 log, bias = 0.017. This performance is to be expected for *external* test molecules, *provided they are adequately represented in the chemical space of the training set.*

#### Big-molecule external test set prediction

Figure 6c illustrates the degree to which the RFR method, trained by small molecules, can predict the solubility of big molecules. The relative accuracy of the prediction (r^2^ = 0.42, RMSE = 1.06 log, MPP = 42%) evidently exceeds that of the GSE and ABSOLV methods (predictive r^2^ = 0 and RMSE > 2 log). To wit, small molecules in the training set provide enough subtle ‘clues’ for the method to extract a sensibly accurate prediction of big-molecule solubility.

The most important descriptors (RDKit terminology – *cf. Abbreviations and definitions*) were found to be *MolLogP >> MolMR > Ipc >> LabuteASA > BertzCT > HeavyAtomMolWt > MolWt > Chi1 > SMR_VSA7 > mp > Chi0v > SMR_VSA10 > PEOE_VSA7 > fr_benzene > Chi1v > E > Chi4v > B*. Some of these are highly intercorrelated. There were additional ~100 descriptors that played lesser and somewhat hidden roles.

In RFR, relationships between descriptors and the model are difficult to extract, and the influence of each compound property on calculated solubility cannot be readily deduced [98]. A major disadvantage to a medicinal chemist is that the RFR result does not directly suggest how compounds could be altered to increase/decrease their solubility. Unlike the intuitive and appealing descriptors in GSE and ABSOLV, many of the RDKit descriptors used are more abstract and not easy to interpret regardless of the modeling method [99].

### Solubility Enhancement–Big Molecules (SEBM)

Table 2 lists the calculated log *S_0_* values of the big molecules. The last column lists the ‘Solubility Enhancement–Big Molecules’ – the ratio of the observed *S_0_* to that calculated by the ABSOLV approach (*cf.*, Eq. 7). The scale quantifies the big-molecule solubility enhancement not predicted by small molecules. A similar ratio using the classic GSE indicates two zones: (a) ‘enhancement’ for compounds to the left of the identity diagonal line in Figure 4b, and (b) ‘attenuation’ for compounds to the right of the line. The GSE zoning is directly linked to the partition coefficient (cf., Fig. 4c). The ABSOLV-based *SEBM* assigns a unified enhancement to all molecules, and separately addresses the role of H-bonding and molecular size (as well as the other Abraham solvation descriptors), whereas the GSE confines the relationship mainly to one descriptor – *clogP*, whose value may not be accurately calculated or measured for large molecules (*e.g.*, ubiquinone).

**Figure 7.**
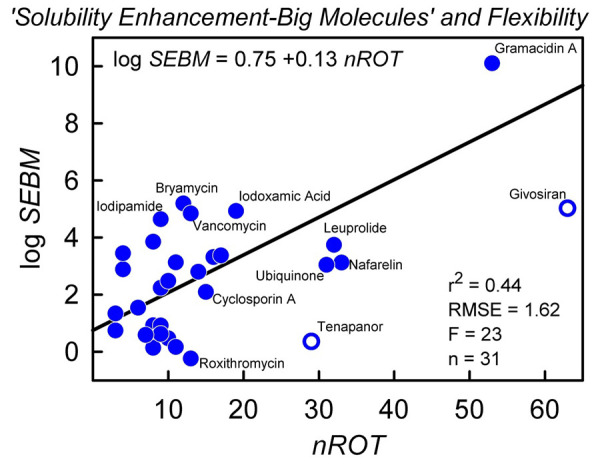
Logarithm of the Solubility Enhancement–Big Molecules as a function of the number of rotatable bonds: log *SEBM* = log *S*_0_^Obs^ – log *S*_0_^ABSOLV^.

Figure 7 is a plot of log *SEBM* as a function of *nROT*. Although noisy, a trend is evident. The unfilled circles in the figure refer to two external test compounds, big molecules recently approved as drugs: givosiran [100] and tenapanor [101], with *M*_W_ 1711 and 1145 Da, respectively.

### Factors that may shed light on the unusual intrinsic aqueous solubility of big molecules

#### Lipophilicity behavior of big vs. small molecules differs

From the GSE analysis, the notable characteristic distinguishing small from big molecules is the dependence on lipophilicity (Fig. 2, Eq. 6b). Big *lipophilic* molecules (ubiquinone, iodipamide, everolimus, telithromycin) are more soluble and big *hydrophilic* molecules (oxytocin, stevioside, nafarelin) are less soluble than predicted by the traditional GSE (*cf*., Fig. 4b). The empirical Eq. (6b) compensates for this tilted relationship with the less negative (-0.4) *clogP* factor and the more negative intercept factor (-1.77) than those in the GSE (-1 and 0.5, resp.), as illustrated in Figure 4c. The solubility-partition correlation using octanol works well for small molecules, but octanol does not appear to match the big-molecule solubility-partitioning behavior in the same way, either because the big molecules are uncharacteristically more soluble in water (extra strong solute-water adhesive interactions) and/or less soluble in the octanol phase (extra strong solute-solute cohesive interactions).

Ermondi *et al.* [27, 28] estimated lipophilicity of nine bRo5 drugs using the well-tested small-molecule ElogP and the new ‘block relevance’ BRlogP chromatographic methods, to investigate the role played by molecular flexibility. They also subjected the molecules to conformational analysis, in order to calculate lipophilicity of various conformers. ElogP chromatographic method appeared to provide an environment in which flexible compounds are driven to assume a more ‘folded’ apolar conformation (as expected in octanol), whereas the BRlogP method favored an ‘extended’ polar conformation for such molecules (as expected in water). Lipophilicity of bRo5 compounds strongly depends on their chameleonic properties: closed form preferred in apolar environments and open form in aqueous media. It is suggested that a non-traditional lipophilicity scale is needed for many bRo5 compounds, which takes into account the solute conformational flexibility and the polarity of the dissolution media [27, 28].

#### Crystal structures of the big molecules and their ‘hydration’ in the solid state

The Appendix shows the 2D structures of the big molecules selected for the study. Many of these are derived from natural products, possessing flexible cyclic and polycyclic components in there structures. The crystal structures of only about half of these molecules have been deposited in the Cambridge Crystallographic Data Centre (CCDC). In many of the compounds the crystal lattices contain *internal* void space that may be filled non-stoichiometrically with water, either fixed at certain positions by H-bonds, or mobile in channels. There are numerous sites with which water could interact by donating H-bonds, possibly competing with acceptor groups in IMHB networks, to form stoichiometric hydrates.

Since most of the crystals chosen for structure determination were grown in semi- or non-aqueous media, the reported X-ray structure of the molecules may not precisely reflect the conformational state found in crystals under conditions where they are equilibrated in aqueous solution, or of dissolved molecules in their unhindered states of hydration. In an exceptional study, the aqueous environment was well mimicked in the synchrotron X-ray determination of the structure of the glycopeptide antibiotic vancomycin [102]. Vancomycin crystals grown by the ‘hanging drop’ method were transferred into a pH 4.6 acetate buffer solution containing 2.2 M NaCl and a cryoprotectant solvent. The suspension was then flash frozen for the low-temperature data collection. The crystal lattice was found to contain an H-bonded dimer of vancomycin, 2 chlorides, 1 acetate, and *105 solvent water molecules* in the asymmetric unit. The organization of the lattice water was not described in the publication.

Zografi and coworkers have conducted pioneering research [103-106] on the influence of adsorbed and absorbed water on the solid state properties of crystalline/amorphous solids, including multicomponent forms such as drug salts and cocrystals. The presence of stoichiometric/nonstoichiometric water in crystalline solids is expected to impact the thermodynamic activity of the solid and thus could affect equilibrium solubility.

#### Enthalpy of solution of big molecules is negative

A computational procedure to normalize solubility data determined at various temperatures to values at a ‘reference’ temperature (*e.g*., 25 °C) was recently described [107]. The enthalpies of solution, Δ*H*_sol_, were predicted from 2D structure, from which the temperature dependence of log *S*_0_ is calculated as:


(8)

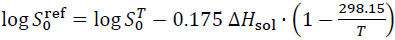



Small molecules, especially weak acids, generally have *positive* enthalpies of solution. For example, naproxen has the calculated Δ*H*_sol_ = +29 kJ/mol. Its solubility at *T* = 310.15 K (37 °C) is log *S*_0_ = -4.03. The value decreases by 0.2 log (Eq. 8) to -4.23 at 25 °C.

Particularly interesting in light of the current study is that just about all the big molecules studied here have *negative* enthalpies of solution (Table 1). The prediction equations [107], based on Abraham descriptors,


(9a)

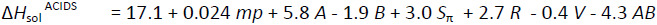




(9b)

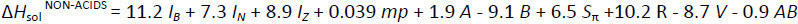



indicate that high H-bond basicity (*B*) and large molar volumes (*V*) correlate with negative (exothermic) enthalpies of solution. Acids (*e.g*., iodipamide and iodoxamic acid) are less inclined to be exothermic, compared to non-acids. (In Eq. (9b), the indicator indices default to zero, except that for a basic molecule, *I*_B_ =1; for a neutral molecule, *I*_N_ = 1; for an ampholyte, *I*_Z_ =1.)

For big basic molecules this means that as temperature rises, the solubility *decreases*. If water is sorbed into the void/channel spaces of crystals containing big molecules, then the negative enthalpy could be rationalized in terms of H-bonding effects. Since water H-bonds weaken with rising temperature, the proportion of the ‘extended’ (water soluble) conformer may shift in favor of the ‘folded’ conformer, which is expected to be less soluble in water. With weakened water binding, the intramolecular H-bond interactions may stabilize the structure in a folded form. In this way, negative enthalpy is consistent with the conformational flexibility of ‘molecular chameleons’ [26-31], and highlights the possible role of sorbed water influences on solubility.

## Conclusion

We have shown that traditional approaches to the prediction of solubility of big molecules (bRO5) do not work very well, unless modified. On the other hand, the RFR method works reasonably well, but it is not easy to understand what specific contributions the various molecular descriptors provide to the overall prediction.

We attempted to link the Solubility Enhancement–Big Molecules (*SEBM*) to other physicochemical properties. A trend was evident in the log *SEBM* vs. *nROT* plot, suggesting that flexibility appears to enhance the solubility of big molecules. In the SGE model, a different lipophilicity scale might improve the performance of the approach, as empirically suggested in Figure 4c and as suggested by the chromatographic studies of Ermondi *et al.* [27, 28]. The introduction of a nonlinear H-bond basicity term in the case of the ABSOLV approach is empirical, and it is not clear how to relate it to first-principle thermodynamic treatment.

Most of the big molecules have negative enthalpy of solution. That is, their solubility *decreases* with increasing temperature. This hints of an important H-bonding role for water sorbed into the solid state of the large molecules. Such molecules appear to have void spaces in their crystal lattices, sufficient to accommodate many water molecules under equilibrium conditions *with crystals wet by aqueous media*.

The observation that the RFR method appears to work encourages us to further search for 3D-based descriptors arising from ‘conformational lipophilicity’ analysis akin to that developed by Caron and coworkers [27-30]. The accurate prediction of the solubility of newly approved molecules originating from the bRo5 chemical space would help in selecting/prioritizing candidates in early drug discovery, particularly if the bRo5 molecular basis of solubility were better understood.

## Abbreviations and definitions

**Table:** 

*S*_0_	“intrinsic” solubility (i.e., the solubility of the *uncharged* form of the compound)
RMSE	root-mean-square error: RMSE = [ 1/n Σ_i_ (y_i_^obs^ - y_i_^calc^)^2^ ]^1/2^, where y^obs^/ y^calc^ = observed/calculated value of log *S_0_* according to model, n = number of measurements of log *S*_0_
r^2^	squared linear correlation coefficient, r^2^ = 1 - Σ_i_ (y_i_^obs^ - y_i_^calc^)^2^ / Σ_i_ (y_i_^obs^ - <y>)^2^ , where y = log *S_0_*, and <y> is the mean value of log *S_0_*
*SD*	standard deviation: *SD* = [ 1/n Σ_i_ (y_i_^obs^ - <y>)^2^ ]^1/2^, where n = number of measurements, <y> = mean value of log *S_0_*
F	F-statistic: F = (n-p-1)/p · Σ_i_ (y_i_^obs^ - <y>)^2^ / Σ_i_ (y_i_^obs^ - y_i_^calc^)^2^, where p = number of regression parameters
MPP	Measure of prediction performance [108]. It refers to the percent of ‘correct’ predictions, as defined by the count of absolute residuals |log *S*_0_^obs^ – log *S*_0_^calc^| ≤ 0.5 divided by the number of measurements. MPP is represented as a pie chart in the correlation plots.

### Abraham solvation descriptors

**Table:** 

*A*	H-bond total acidity
*B*	H-bond total basicity
*S_π_*	dipolarity/polarizability due to solute-solvent interactions between bond dipoles and induced dipoles
*E*	excess molar refraction (dm^3^ mol^-1^ / 10); which models dispersion force interaction arising from π- and n-electrons of the solute
*V*	McGowan molar volume (dm^3^ mol^-1^ / 100)
*A·B*	acid-base H-bonding product descriptor used in ABSOLV solubility prediction

### Most important RDKit descriptors in RFR analysis

#### Subdivided Surface Area Molecular Descriptors [109]

**Table:** 

*LabuteVSA*	sum of atomic contributions [110] to the accessible van der Waals surface area
*MolLogP*	sum of atomic contributions to octanol/water partition coefficient, log *P*
*MolMR*	sum of atomic contributions to molar refractivity, *MR*
*SMR_VSAk*	sum of accessible van der Waals surface area for those atoms with atomic contribution to molar refractivity; k refers to a small domain of atomic-contribution to MR; intended to capture *molecular size & polarizability*
*PEOE_VSAk*	intended to capture *direct electrostatic interactions* in a particular range; based on iterative equalization of atomic *orbital electronegativities* [111].

#### Complexity descriptors

**Table:** 

*BertzCT*	complexity index, based on size, symmetry, branching, rings, multiple bonds, and heteroatoms characteristic of solute [112].
*Ipc*	content information of topological graph [113] - entropy of atomic distribution in solute

#### Topological and electrotopological connectivity indices

*Chi0, Chi0n, Chi0v, Chi1, Chi1n, Chi4n, Chi4v, α* – Kier-Hall topological connectivity and shape indices [114, 115]; numerical representations of topology of solute calculated from graphical depiction of the molecule

Atomic and subroup counts, *HeavyAtomCount, NumberAromaticCarbocycles, NumberAromaticRings, RingCount, fr_benzene*

### Availability of the *Wiki-pS_0_* Database

The entire *Wiki-pS_0_* database is planned to be released in book form: A. Avdeef. *Intrinsic Aqueous Solubility Data for Pharmaceutical Research.* Wiley-Interscience, Hoboken, NJ (under discussion with publisher). A sampling is presented in Table A5 in [20].

## Figures and Tables

**Table 1.** Big-molecule (bRo5) physicochemical properties

Compound	*log S_0_*	SD	n	*M*_W_	*mp*	*clogP*	Δ*H*^0^_sol_	*NHA*	*NHD*	*nROT*	*A*	*B*	*S_π_*	*E*	*V*	Ref
Amphotericin B	-3.52	0.69	2	924	179	0.71	-33	17	12	3	3.55	5.99	5.12	4.47	7.12	[63, 64]
Anidulafungin	-4.36	0.45		1140	250	-0.93	0	17	14	14	3.67	8.22	10.4	7.8	8.4	[63]
Bryamycin	-4.14	0.29	3	1665	210	0.77	-16	31	17	12	4.47	11.56	14.55	10.51	11.65	[64-66]
Cyclosporine A	-5.03	0.16	6	1203	151	3.27	-40	12	5	15	1.25	7.61	10.16	4.23	10.02	[63, 67-69]
Dactinomycin	-3.22	0.16	2	1255	242	0.73	-10	18	5	8	1.36	8.49	11.45	6.12	9.49	[70]
Docetaxel	-5.14	0.05	2	808	232	3.26	-12	14	5	8	1.03	4.01	4.18	3.47	5.92	[63, 71]
Everolimus	-5.02	0.58		958	138	6.20	-36	14	3	9	0.63	4.73	4.73	3.29	7.68	[63, 72]
Gramicidin A	-4.16	0.41		1882	229	4.37	-31	17	21	53	5.57	11.3	17.8	10.26	14.81	[73]
Gramicidin S	-3.89	0.35		1141	169	1.23	-14	12	10	16	2.46	7.42	10.69	5.38	9.12	[64]
Iodipamide	-5.47	0.67		1140	307	6.85	45	4	4	9	2.25	1.87	4.84	5.86	4.38	[50]
Iodoxamic Acid	-5.49	0.36		1288	224	6.13	35	8	4	19	2.25	2.73	5.48	6.01	5.46	[74]
Ivermectin	-5.56	0.39	5	875	140	5.60	-33	14	3	8	0.68	4.23	3.21	3.24	6.72	[72, 75-78]
Leuprolide	-3.15	0.20		1209	153	-1.44	-11	14	15	32	4.25	8.66	11.75	7.23	9.21	[63]
Nafarelin	-5.61	0.52		1323	189	-1.62	2	15	16	33	4.74	9.32	13.46	8.93	9.88	[79]
Nystatin	-4.1	0.39		926	170	0.94	-35	17	12	3	3.55	5.93	5.02	4.32	7.16	[80]
Oxytocin	-1.2	0.17		1007	164	-3.61	2	15	12	17	3.96	7.67	11.34	5.9	7.47	[81]
Paclitaxel	-6.53	0.14	2	854	216	3.74	-3	14	4	10	0.9	4.13	5.22	4.05	6.2	[82, 83]
Paclitaxel analog12	-5.48	0.67		808	187	3.20	-14	14	5	8	1.03	4.01	4.18	3.47	5.92	[71]
Paclitaxel analog17	-4.52	0.48		802	179	3.32	-24	14	5	9	1.03	3.97	3.67	2.87	6.02	[71]
Paclitaxel analog23	-5.78	0.73		807	187	3.23	-13	13	6	8	1.33	4.15	4.31	3.63	5.96	[71]
Rapamycin	-5.55	0.69		914	184	6.18	-30	13	3	6	0.63	4.51	4.57	3.26	7.34	[84]
Rifabutin	-4.09	0.66	3	847	176	4.62	-9	14	5	4	1.31	4.39	4.43	4.24	6.47	[63, 85, 86]
Rifampicin	-2.96	0.27	9	823	164	4.34	-6	15	6	4	2.55	4.66	4.67	4.73	6.21	[87-91]
Roxithromycin	-3.98	0.37		837	120	2.21	-47	17	5	13	1.05	5.12	2.9	2.58	6.55	[63]
Solithromycin	-6.23	0.14		845	189	4.60	-12	15	2	11	0.35	4.46	4.7	3.67	6.44	[92]
Stevioside	-2.83	0.17		805	198	-2.94	-21	18	11	9	2.74	5.49	4.29	4.25	5.67	[93]
Tacrolimus	-5.42	0.54	2	804	126	4.64	-27	12	3	7	0.71	3.98	3.98	2.82	6.38	[63, 94]
Telithromycin	-3.02	0.17		812	188	4.93	-13	14	1	11	0.12	4.40	4.53	3.49	6.32	[63]
Temsirolimus	-5.06	0.59		1030	134	5.72	-39	16	4	10	1.02	5.07	5.25	3.46	8.17	[63]
Ubiquinone	-7.56	1.65	2	863	48	17.85	-53	4	0	31	0.00	2.20	1.16	2.15	7.95	[95, 96]
Vancomycin	-2.13	0.14		1449	175	0.11	-22	25	19	13	5.81	10.56	12.32	9.73	9.88	[97]

^a^ log S*_0_* averaged for n > 1 sources (references in last column). SD is the estimated standard deviation in the measured value. *ΔH*^0^_sol_ (kJ/mol) are calculated enthalpies of solution (see text). *nROT* is the number of rotatable bonds in the molecule. For the other terms, *cf*. *Abbreviations and definitions*.

**Table 2.** Calculated log *S_0_* and 'Solubility Enhancement-Big Molecules'

Compound	*Obs*	GSE ^a^	ABSOLV ^b^	RFR ^c^	SEBM ^d^
Amphotericin B	-3.52	-1.75	-4.86	-3.25	22
Anidulafungin	-4.36	-0.82	-7.16	-4.30	628
Bryamycin	-4.14	-2.12	-9.33	-3.90	156099
Cyclosporine A	-5.03	-4.03	-7.12	-4.65	123
Dactinomycin	-3.22	-2.40	-7.07	-3.82	7080
Docetaxel	-5.14	-4.83	-5.97	-4.86	7
Everolimus	-5.02	-6.83	-7.25	-5.93	172
Gramicidin A	-4.16	-5.91	-14.26	-6.00	12667592811
Gramicidin S	-3.89	-2.17	-7.20	-3.78	2056
Iodipamide	-5.47	-9.17	-10.11	-6.51	43888
Iodoxamic Acid	-5.49	-7.62	-10.42	-5.96	85035
Ivermectin	-5.56	-6.25	-6.48	-5.92	8
Leuprolide	-3.15	0.67	-6.89	-3.84	5514
Nafarelin	-5.61	0.48	-8.73	-4.24	1314
Nystatin	-4.10	-1.89	-4.84	-3.16	5
Oxytocin	-1.20	2.72	-4.57	-2.87	2350
Paclitaxel	-6.53	-5.15	-7.00	-5.67	3
Paclitaxel analog12	-5.48	-4.32	-5.97	-4.84	3
Paclitaxel analog17	-4.52	-4.36	-5.44	-4.61	8
Paclitaxel analog23	-5.78	-4.35	-5.91	-4.98	1
Rapamycin	-5.55	-7.27	-7.09	-6.05	35
Rifabutin	-4.09	-5.63	-6.97	-5.65	765
Rifampicin	-2.96	-5.23	-6.40	-5.47	2780
Roxithromycin	-3.98	-2.66	-3.74	-3.76	1
Solithromycin	-6.23	-5.74	-6.39	-5.76	1
Stevioside	-2.83	1.71	-3.45	-2.22	4
Tacrolimus	-5.42	-5.15	-6.01	-5.53	4
Telithromycin	-3.02	-6.06	-6.15	-5.50	1340
Temsirolimus	-5.06	-6.31	-7.54	-5.97	301
Ubiquinone	-7.56	-17.58	-10.60	-6.59	1102
Vancomycin	-2.13	-1.11	-6.97	-4.01	69548

^a^ Calculated log *S*_0_ in Fig. 4b.

^b^ Calculated log *S*_0_ in Fig. 5b.

^c^ Calculated log *S*_0_ in Fig. 6c.

^d^ Observed *S_0_* divided by the value calculated in ABSOLV analysis: SEBM = *S*_0_^Obs^/*S*_0_^ABSOLV^.

## Appendix

Underlined names denote compounds whose crystal structures have not been deposited in the Cambridge Crystallographic Data Centre.
